# Two-Phase Framework for Indoor Positioning Systems Using Visible Light †

**DOI:** 10.3390/s18061917

**Published:** 2018-06-12

**Authors:** Gregary B. Prince, Thomas D. C. Little

**Affiliations:** Department of Electrical and Computer Engineering, College of Engineering, Boston University, Boston, MA 02215, USA; tdcl@bu.edu

**Keywords:** indoor positioning, RSS, AoA, ToF, trilateration, triangulation, proximity, FDMA, CDMA, TDMA, OFDM, PPM, OOK, PAM, visible light

## Abstract

Recently with the advancement of solid state lighting and the application thereof to Visible Light Communications (VLC), the concept of Visible Light Positioning (VLP) has been targeted as a very attractive indoor positioning system (IPS) due to its ubiquity, directionality, spatial reuse, and relatively high modulation bandwidth. IPSs, in general, have four major components: (1) a modulation, (2) a multiple access scheme, (3) a channel measurement, and (4) a positioning algorithm. A number of VLP approaches have been proposed in the literature and primarily focus on a fixed combination of these elements and moreover evaluate the quality of the contribution often by accuracy or precision alone. In this article, we provide a novel two-phase indoor positioning algorithmic framework that is able to increase robustness when subject to insufficient anchor luminaries and also incorporate any combination of the four major IPS components.

## 1. Introduction

Indoor positioning has been explored in many contexts, but most often finds application in piggybacking existing communication infrastructures (e.g., WiFi, Bluetooth) due to the fact that customized solutions (e.g., ultrawideband (UWB), Ultrasound, and IR) require hardware devoted solely to positioning in addition to existing communication infrastructure it could be deemed as too expensive of an investment for some indoor spaces. Moreover, positioning in the conventional sense requires transceivers capable of processing channel measurements (e.g., angle, signal strength, time of flight) and relate them to positional estimates through positioning algorithms (e.g., multilateration, triangulation). With the transition from electric to electronic lighting and the ubiquity of visible light (via Luminaries) in indoor spaces, visible light may prove to be the next communication infrastructure to exploit for positioning services. Visible light is one of the most viable solutions to indoor positioning due to its directionality, short impulse response, and the distribution and availability of anchor (beacon) luminaries to meet the illumination needs of indoor spaces, while its issues to overcome are installation accuracy, network layer identification, coexistence of WiFi and visible light communication (VLC), and efficient multiple access schemes. The work in the visible light positioning area is fairly new comparatively, thereby providing the opportunity for many avenues to still be explored. Despite being new, there has been an effort to define international standards for the adoption of visible light positioning while maintaining the integrity of illumination and not necessarily VLC (JEITA CP-1222) [1].

Device positioning using visible light is not yet a rich field of research, compared to those in the RF spectrum. The investigations to date can be organized into three groups: (1) approaches which depend on the current lighting infrastructure (e.g., fluorescent lighting), (2) approaches which depend on imaging, and (3) those which look forward into the LED installation assumption. There are four essential components to visible light positioning systems: (1) Modulation, (2) Multiple Access, (3) Measurement, (4) Positioning Algorithm. State of the art surveys on the topic of indoor positioning systems using visible light communication, or otherwise referred to as visible light positioning (VLP), generally outline the specific components that are possible to use in such a system [2,3]. This work does not aim to produce another survey nor a new VLP system, but rather discusses how the four aforementioned VLP components can be integrated into the Two-Phase framework in a broader sense than the initial published work that focused on the OOK, TDM, AOA, Optimization combination [4].

## 2. Visible Light Positioning Systems

In its infancy, researchers investigated the application of existing fluorescent lighting to establish positioning services [5,6]. These solutions have shown promise for positioning, but are highly constrained in terms of communication data rate, achieving maximally 2.5 kbps. Given the recent advancement and the energy efficient lighting replacement solution the light emitting diode (LED) has been given coupled with its high switching speed (20 MHz) researchers have been looking into how LED lighting may be exploited. It should be noted that the dual-use focus of LED lighting has been predominately on high throughput spatial reuse communication channels, but there have been a fraction thereof related to positioning [7]. In [8], the fundamentals of the LED communication channel with perfect transmitter receiver alignment are provided and illustrates accuracies on the order of centimeters. Other works such as [9,10] investigates the accompanying of a receiver with a 6 axis sensor to accommodate for orientation imperfections. Receiver position estimation without requiring height knowledge and LOS alignment without specific knowledge of the transmit LEDs physical parameters is investigated in [11,12].

Whereas [13] reinforces the cost, multipath, and latency problems with existing approaches and introduces a unique approach to determining the location of a target in three dimensions. It is accomplished in two phases: firstly, by estimating the target location in a two dimensional plane (x,y) and then secondly, by layering the height via a minimization to determine (z). This is accomplished by requiring a very strict inflexible grid layout. Another positioning scheme in which the estimates of the coordinates in the (x,y) plane and the height (z) coordinate are estimated sequentially is introduced in the works of [14] using RSS. This work improves upon just acquiring a location estimate by employing tracking using sequential importance sampling and Kalman Filtering with reported accuracies ranging from 9–15 cm. The works of [15] relies on more than the minimum required number of signals typically required for trilateration and determining the possible roots of the overdetermined range equations. This method also computes (x,y) and then (z). The works of [16] investigates and proposes a weighting factor based on RF carrier allocation [17,18] be applied to compensate for the effect of tilt at the receiver, for which the motivation is the VLC investigations simplify the problem by assuming the transmitter and receiver are parallel. The investigation [19] establishes a study with a particular frame structure with 10 nanosecond bit periods and 50 nanosecond guard periods to guarantee distinctive reception of pilot tones. Cross correlation is performed with the pilot train to determine the tone of interest, this frame structure and correlation is performed with no consideration provided to any other communication or illumination levels. This method encourages monitoring power levels of pilot tones exclusively absent other communications rather than relying on VLC to communicate unique IDs of the anchor luminaries while also ignoring the estimate of the height (z) of the target.

The works of [20] also consider the imposition of choking the data rate allowed by imposing a basic frame slotted ALOHA frame structure. A concept of effective positional radius is developed and applied to relatively simple geometric configurations with both moving and static objects [21]. A TDoA approach using phase difference measurement where unique frequency ID’s are assigned to each luminaire. Despite sub-centimeter results the use of frequency IDs can impact the quality of light as well as the VLC communication capability and added complexity in the receiving device (e.g., bank of band-pass filters) [22,23]. Another option that has been investigated is the application of Dual Tone Multi-Frequency (DTMF) Techniques, which are employed in the phone system, to VLC based positioning [24]; it does not require clock synchronization to distinguish signals spanning time-slots and encodes each luminaire with an *N* digit ID in DTMF signal. Conventional lighting uses Pulse Position Modulation (PPM) or On Off Keying (OOK) modulation not DTMF and could have an impact to lighting quality. A positioning technique based on transmitting sinusoidal components is provided [25,26]. They define a simple hybrid RF-VLC positioning approach using range-free VLC (proximity) sensing and fuses with RF RSSI measurements.

Different ranging signals are a topic to investigate for consideration of other constraints such as illumination and communication [27]. The technique of fingerprinting using LEDs has also been given consideration for such indoor positioning systems [28]. A robust and low complexity self-localization method, which does not require synchronization and can also accommodate intermittent LOS blockages, is described using Bayesian signal models [29]. Each luminaire again has a unique identifier and this work assumes that the target knows the positions of the beacons across an entire floor layout of a building rather than confined to a single room. A received signal strength ratio method is introduced in which, assuming optical power from each luminaire is separable, the ratio of LOS distances can be obtained. It is assumed the height is known, and that the reflective components of the channel are not relevant. A simple set of circular and linear equations are solved to produce a two dimensional position estimate with accuracy of 3.24 cm [30,31,32]. A scene analysis method using white LEDs is proposed in [33] and utilizes the RSS information from four LED luminaries as fingerprints for different locations with an accuracy of 0.65 cm.

The concept of combining various sources of measurements (modalities) as complimentary to a core technology or as a failsafe backup for positioning or communication performance is not a novel concept (i.e., combining ultrasound with WiFi) [34,35,36]. In [37] it was proposed and experimentally demonstrated a proximity-based visible light indoor positioning system provided that uses a Zigbee wireless network to distribute location information to the main node and extend the footprint of the network. As the literature demonstrates, there are many approaches to solve the problem of indoor positioning using visible light as a medium. The work presented in this manuscript does not directly aim to compete by solely developing either a new positioning algorithm that outperforms another by a relative amount, a new modulation format, a new multiple access system, or a novel measurement, but rather takes a system view at the problem that can incorporate combinations of the best approaches in the state of the art based on requirements or constraints of an implementation. In this work we provide a different context through the development of *A Two-Phase Positioning Framework*.

## 3. Two-Phase Positioning Framework

There are several major factors to consider prior to the adoption of a positioning technology: (1) Accuracy, (2) Precision, (3) Robustness, (4) Scalability, (5) Complexity, and (6) Cost. We propose an positioning framework that can take advantage of ubiquity, directionality, timeliness, blockage conditions, and measurement quality to provide positional estimates under an array of scenarios and constraints. Typical positioning systems require multiple reference “beacons” (referred to as Luminaries in the rest of this manuscript) to simply produce a positional estimate. The framework has two phases; the *coarse phase* produces a weighted proximity estimate with as few as one Luminaries within a mobile terminal’s (MTs) field of view (FoV), and a *fine phase*, in which conventional positioning algorithms can be performed if sufficient Luminaries are within the MTs FoV. Moreover, Fine phase positioning using many channel measurements to perform lateration or angulation requires more computation for improved accuracy whereas coarse positioning can provide position estimate within with relatively minimal computation. Positioning systems also typically rely on access to sufficient anchor landmarks to arrive at the positional estimate (often at least three). Under blockage conditions, access to sufficient landmarks is often jeopardized and conventional approaches cannot arrive at an estimate. Furthermore, when reflections and multipath arise, the measurements are too poor to produce a proper estimate. It is shown that from a MT perspective, integrating the two stages of coarse and fine positioning in an intelligent fashion can dynamically balance between the needs of positioning performance across the major factors impacting the adoption of a positioning technology. Instead of solely relying on triangulation or trilateration, the MT’s estimate their locations firstly by exploiting received optical power level observables with unique IDs via a chosen channel multiplexing scheme to establish a coarse estimate, and secondly use either the azimuth and elevation Angle of Arrival (AoA), or N≥3 bearing AoA, RSS, and/or ToF measurements to establish a fine estimate via triangulation or trialteration respectively or any appropriate positioning algorithm for that matter. In many cases, the fine estimate will improve upon the coarse estimate; however when multi-Luminaire positioning fails, this framework yields the coarse estimate rather than a positioning failure. Since the environment model relies on the primary requirement of adequate illumination, the number of LED anchors, and transmit power for communication functions are determined.

### 3.1. Model Framework

**Figure 1:**
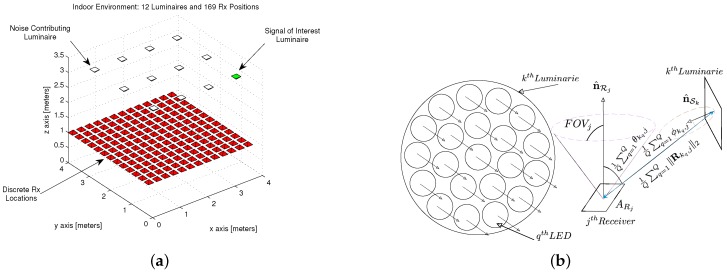
Model Description: (**a**) Indoor Environment Model with 12 Source Luminaries and 169 Discrete Receiver Positions in a 4 m by 4 m by 3.5 m room without Obstructions; and (**b**) iVisible Light Channel Geometry Model for the *k*th source Luminaire with *Q* LED’s Transmitting Modulated Data to the *j*th Receiver in an Indoor Space.

The CandLES simulation environment [38] is used to model an example 4 by 4 by 3.5 m room, with twelve (12) anchor luminaries mounted to the ceiling and receivers at varying discrete positions within the room. Figure 1a provides a view of the indoor environment model using CandLES. The simulation parameters are provided in Table 1. The model measures the system impulse response, noise levels, as well as received optical power and reception angles at each of the discrete receiver locations from each of the twelve luminaire anchors by employing various multiplexing schemes to the visible light channel such as TDM, FDM, and CDM The collection of these measurements within this indoor space are used as input to the proposed two phase framework herein.

To provide a theoretical analysis of the performance of the two-phase framework [4] we extend the typical point source analysis for a luminaire to account for multi-source combination and reflections that lead to temporal and angular dispersion in the impulse response $h(t,\phi_{k_{q},j},\theta_{k_{q},j})$ between sources (anchor luminaries) $S_{k_{q}}$ and receivers (VLC-capable MT) $R_{j}$ in Equation (1). Note that δ(t−a) is the Dirac delta function, which is assigned the value 0 at all points in the domain except for at the value t=a, where it is assigned the value 1.

(1) $$h_{S_{k_{q}},R_{j}}\left(t,\phi_{k_{q},j},\theta_{k_{q},j}\right)=H_{f}^{k_{q},j}\left(0,\phi_{k_{q},j},\theta_{k_{q},j}\right)\delta \left(t-\frac{{∥R_{k_{q},j}∥}_{2}}{c}\right)$$

The $H_{f}^{k_{q},j}\left(0,\phi_{k_{q},j},\theta_{k_{q},j}\right)$ term in the impulse response is termed the DC channel gain due to its constant effect on the received optical power over the visible light channel. Equations (2) and (3) illustrate the geometric and device parameter influence on the DC channel gain.

(2) $$H_{f}^{k_{q},j}\left(0,\phi_{k_{q},j},\theta_{k_{q},j}\right)=\frac{\left(m_{k_{q}}+1\right)A_{R_{j}}}{2\pi {∥R_{k_{q},j}∥}_{2}^{2}}\cos^{m_{k_{q}}}\phi_{k_{q},j}\cos\theta_{k_{q},j}\Pi \left(\frac{\theta_{k_{q},j}}{FOV_{j}}\right)$$

(3) Πxa=−1ifx≤a−0ifx>a

Another way to interpret (2) is a product of the transmitted radiation pattern $Tx\left(\phi_{k_{q},j}\right)=\frac{\left(m_{k_{q}}+1\right)}{2\pi }\cos^{m_{k_{q}}}$$\left(\phi_{k_{q},j}\right)$ and a receiver response $Rx\left(\theta_{k_{q},j}\right)=A_{R_{j}}\cos\left(\theta_{k_{q},j}\right)\Pi \left(\frac{\theta_{k_{q},j}}{FOV_{j}}\right)$ over the square of the distance traveled ${∥R_{k_{q},j}∥}_{2}^{2}$

(4) $$H_{f}^{k_{q},j}\left(0,\phi_{k_{q},j},\theta_{k_{q},j}\right)=\frac{Tx\left(\phi_{k_{q},j}\right)Rx\left(\theta_{k_{q},j}\right)}{{∥R_{k_{q},j}∥}_{2}^{2}}$$

Figure 1b illustrates the geometric building blocks of the source Luminary - receiver pair over the visible light channel. The vector between the source Luminary LED’s and the receiver is given by $R_{k_{q},j}$ in Equation (5).

(5) $$R_{k_{q},j}=r_{S_{k_{q}}}-r_{R_{j}}=\left[\begin{array}{c} x_{k_{q}}-x_{j}\\ y_{k_{q}}-y_{j}\\ z_{k_{q}}-z_{j} \end{array}\right]$$

The free space range delay between each LED’s in the source Luminary and the receiver is simply the $l_{2}$ norm given by Equation (6). The centroid of the LED’s in the *k*th source Luminary with *Q* LEDs is given by Equation (7).

(6) $${∥R_{k_{q},j}∥}_{2}=\sqrt{{\left(x_{k_{q}}-x_{j}\right)}^{2}+{\left(y_{k_{q}}-y_{j}\right)}^{2}+{\left(z_{k_{q}}-z_{j}\right)}^{2}}$$

(7) $${∥R_{k,j}∥}_{2}=\frac{1}{Q}\sum_{q=1}^{Q}{∥R_{k_{q},j}∥}_{2}$$

The impulse response of the channel is provided by (1) and (2) with Lambertian pattern order $m_{k_{q}}$, receiver area $A_{R_{j}}$, angle of irradiance $\phi_{k,j}=\frac{1}{Q}\sum_{q=1}^{Q}\phi_{k_{q},j}$ from the *q*th LED within the *k*th luminaire to the *j*th receiver.

The Lambertian order $m_{k_{q}}$, is a function of the the half angle semi-power, $\Phi_{\frac{1}{2}}$ of the sources emission. The higher the order, the more narrow and directed the emission becomes, while as it decreases it the emission becomes broad.

(8) $$m_{k_{q}}=-\frac{\ln2}{\ln(\cos\left(\Phi_{\frac{1}{2}}\right))}$$

The angle of irradiance is the $\phi_{k_{q},j}$ is the angle between the normal of the LED source ${\hat{n}}_{S_{k_{q}}}$ and the line of sight vector between the LED source and photodetector receiver $\frac{-r_{S_{k_{q}}}+r_{R_{j}}}{{∥R_{k_{q},j}∥}_{2}}$.

(9) $$\cos\left(\phi_{k_{q},j}\right)={\hat{n}}_{S_{k_{q}}}\cdot \frac{-r_{S_{k_{q}}}+r_{R_{j}}}{{∥R_{k_{q},j}∥}_{2}}=-\frac{{\hat{n}}_{S_{k_{q}}}^{T}R_{k_{q},j}}{{∥R_{k_{q},j}∥}_{2}}$$

The angle of incidence is the $\theta_{k_{q},j}$ is the angle between the normal of the photodetector receiver ${\hat{n}}_{R_{j}}$ and the line of sight vector between the LED source and photodetector receiver $\frac{r_{S_{k_{q}}}-r_{R_{j}}}{{∥R_{k_{q},j}∥}_{2}}$.

The angle of incidence can be averaged within a single luminaire with *Q* LEDs: $\theta_{k,j}=\frac{1}{Q}\sum_{q=1}^{Q}\theta_{k_{q},j}$ received by the *j*th receiver from the *q*th LED within the *k*th luminaire, and receiver field of view is $FOV_{j}$ [8].

(10) $$\cos\left(\theta_{k_{q},j}\right)={\hat{n}}_{R_{j}}\cdot \frac{r_{S_{k_{q}}}-r_{R_{j}}}{{∥R_{k_{q},j}∥}_{2}}=\frac{{\hat{n}}_{R_{j}}^{T}R_{k_{q},j}}{{∥R_{k_{q},j}∥}_{2}}$$

The orientations of the anchor Luminaries sources and MT receivers are ${\hat{n}}_{S_{k}}$ and ${\hat{n}}_{R_{j}}$ respectively.

### 3.2. Coarse Phase

The *coarse* phase of the two-phase framework depends heavily on the infrastructure layout as well as the modulation and multiplexing schemes employed of the luminaire anchors. Prior positioning approaches in the context of sectors or cells has been investigated [39]. These approaches are able to bound the worst case positioning error to the radius of the sector itself through code signaling or in-range sensing. The context of WiFi/VLC cooperative localization [36] is also considered within an indoor environment, equipped with LED luminaries designed and distributed in a manner such that the illumination requirement is satisfied.

The implication of the illumination requirement from a communications perspective, simply guarantees connectivity over the majority of the indoor environment however the rate at which communications may occur can be limited by illumination levels despite having ubiquitous sources. Whereas the implication from a positioning perspective is luminaire anchors supplying sufficient optical power to enable signal demodulation at the MT to determine ToF, RSS, TDoA, AoA, etc., above ambient noise levels. The *k*th luminaire is assumed to be installed at a position $r_{S_{k},\mathrm{desired}}$ in the relative coordinate frame along with its relative orientation ${\hat{n}}_{S_{k},\mathrm{desired}}$; however these absolute positions and orientations are subject to installation errors $\left(r_{S_{k_{,\mathrm{offset}}}},{\hat{n}}_{S_{k_{,\mathrm{offset}}}}\right)$, which are modeled as Additive White Gaussian Noise (AWGN) in this article.

(11) $$r_{S_{k_{q,\mathrm{actual}}}}=r_{S_{k_{q,\mathrm{desired}}}}+r_{S_{k_{q,\mathrm{offset}}}}\;,\;\forall k\in L\;,\;\forall q\in Q$$

(12) $${\hat{n}}_{S_{k_{q,\mathrm{actual}}}}={\hat{n}}_{S_{k_{q,\mathrm{desired}}}}+{\hat{n}}_{S_{k_{q,\mathrm{offset}}}}\;,\;\forall k\in L\;,\;\forall q\in Q$$

**Figure 2:**
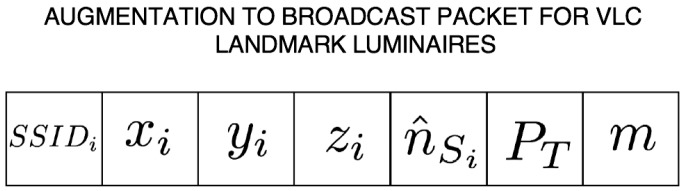
Broadcast VLC Packet.

Furthermore, the coarse algorithm requires multiple access to the VLC channel; this may be accomplished via time division multiplexing (TDM), frequency division multiplexing (FDM), code division multiplexing (CDM), wavelength color division multiplexing (WCDM), or even space division multiplexing (SDM) to ensure MT association with distinct anchor Luminaries in the field of view as discussed in Section 3.2.2 of this article, we focus on TDM, FDM, and CDM. At the network layer each landmark luminaire is required to have its own unique ID (SSID), and the capability to packetize the position, orientation, transmit optical power, and beam pattern mode as outlined in the Figure 2.

The MT has the capability to process the network layer packets and measure the received optical power for each luminaire transmission in the multiplexing scheme and report an estimate when subject to the environment. The principle idea is that prior to establishing a communication connection, the device can broadcast its presence to the infrastructure. The coarse estimate is obtained as a weighted positional proximity estimate whose weights are the measured optical power from each luminaire. Additional binary weights $\epsilon_{k_{q}}\in 0,1$ are applied to the received optical power to discount outliers in the observations, which can occur at varying orientations. Given adequate illumination levels a 10 dB outlier threshold was used. Equation (13) outlines the coarse weighted estimate of device position.

(13) $${\hat{r}}_{R_{j_{COARSE}}}=\frac{\frac{1}{Q}\sum_{q=1}^{Q}\sum_{k=1}^{L}\epsilon_{k_{q}}P_{R_{k_{q},j}}\left(\phi_{k_{q},j},\theta_{k_{q},j}\right)r_{S_{k_{q,\mathrm{desired}}}}}{\frac{1}{Q}\sum_{q=1}^{Q}\sum_{k=1}^{L}\epsilon_{k_{q}}P_{R_{k_{q},j}}\left(\phi_{k_{q},j},\theta_{k_{q},j}\right)}$$

**Figure 3:**
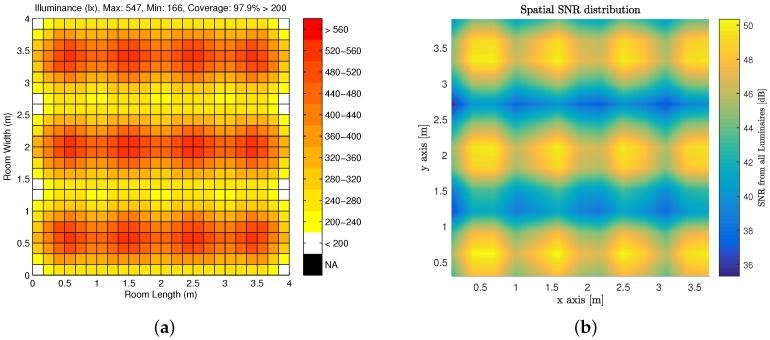
Model Description: (**a**) Illumination Pattern for Candidate Indoor Environment; and (**b**) Aggregate SNR Distribution over Candidate Indoor Environment for a $R_{b}=20$ Mbps.

The illumination pattern as well as the SNR provided over the entire indoor simulation space are shown in Figure 3a,b respectively. One can observe that there is sufficient illumination and by consequence sufficient SNR throughout the entire room to perform human functions and electronics functions respectively.

One of the largest benefits of this approach is that simply observing the brightest luminaire does not yield an appropriate feasible estimate of position, especially when multiple luminaries are within the FoV of the MT. The *coarse* phase concludes by returning the other visible light channel measurements it observes and if a sufficient number of anchor luminaries are in view then they are used in the *fine* phase. A large benefit of the two-phase framework is its ability to balance the needs of a particular use case. Certain use cases require a rapid positional estimate of a lower grade accuracy, whereas other user cases may require the highest grade accuracy estimate and can also accommodate the time required. Another benefit of this framework is its [4] ability to always produce a positional estimate, albeit at different accuracies and the quality of such an estimate over the whole footprint of an indoor space. This guarantee of an estimate is based on the principle that luminaire projects an illumination pattern of a certain radius, with its center being the luminaire anchored position, on the floor. This provides a robustness aspect to adopt this two-phase framework. Based on the lighting design the radii of the illumination floor projections may or may not overlap.

Proximity methods are the most simple as they do not require triangulation, trilateration, or ingerprinting to produce an absolute or relative positional estimate, but can still often yield very useful results. In visible light positioning systems the proximity information is typically acquired through identification codes that are transmitted from each LED luminary and correlated with a database when a mobile terminal receives it while being underneath its illumination pattern. Some of the key hurdles to overcome are establishing a mechanism to communicate the discovered IDs back to a centralized database on the uplink, determining a method to locally correlate IDs to an overlaid map within the mobile terminal, minimizing the impact of the downlink communication overhead required to transmit the IDs continuously but provide enough information to arrive at a positional estimate albeit a second stage of estimation e.g., lateration algorithms. With the aid of other onboard sensors, the mobile terminal can reduce the uncertainty region of the entire illumination footprint from the corresponding ID to a more narrow segment of the illumination footprint.

(14) $${\left[\hat{x},\hat{y},\hat{z}\right]}^{T}=\min_{r}f\left(\hat{r},r\right)$$

#### 3.2.1. Modulations

##### Pulsed Modulation

**Figure 4:**
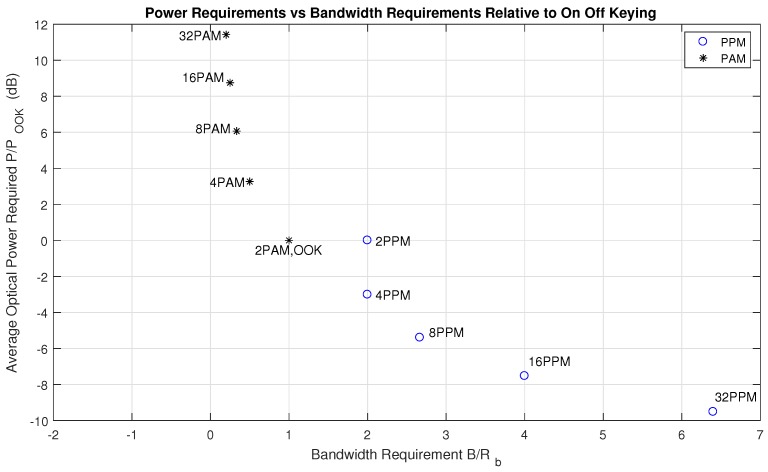
Pulsed Modulations Illustrating Power and Bandwidth Tradeoffs for L-PPM and M-PAM relative to OOK [38].

We inspect OOK as a baseline modulation standard for signaling over the visible light channel. Figure 4 illustrates the relative power requirement increases relative to OOK for M-PAM modulations and the relative bandwidth requirement increases to OOK for L-PPM.

The relative optical power penalty for M-PAM relative to OOK is:

(15) $$\frac{P_{MPAM}}{P_{OOK-NRZ}}=10\log_{10}\left(\frac{M-1}{\sqrt{\log_{2}\left(M\right)}}\right)$$

The relative bandwidth penalty for L-PPM relative to OOK-NRZ is:

(16) $$\frac{B_{LPPM}}{B_{OOK-NRZ}}=\frac{\frac{LR_{b}}{\log_{2}\left(L\right)}}{R_{b}}=\frac{L}{\log_{2}\left(L\right)}$$

In this article, we evaluate positioning performance using OOK-NRZ, 2-PPM, and 4-PAM pulsed modulation formats. 2-PPM requires double the bandwidth and the same optical power to transmit the same bit rate as OOK-NRZ and 4-PAM requires 3.3 dB more optical power to transmit the same bit rate over the same bandwidth.

##### Muti-Carrier Modulations

Just as in RF channels, the requirement to fit more data into a given channel efficiently gave rise to investigations into more clever modulation and multiple access schemes. OFDM when applied in RF channels is a bipolar waveform. When applied to an IM-DD Optical channel the negative portion of the waveform needs to biased or zeroed. There are many approaches that have been proposed to eliminate the negative portion of the waveform in an efficient manner as to minimally impact the communication performance of the scheme in the optical channel. This section outlines the major modifications to OFDM for suitable use in the optical channel.

In efforts to increase data rates even further than pulsed baseband modulation schemes can offer, OFDM schemes have been explored to be modified to meet the Optical Wireless Channel constraints. There are two major approaches evaluated in this article to adapt OFDM to the OW Channel, despite numerous other variations on OFDM: DC - biased Optical OFDM (DCO-OFDM) and Asymmetrically Clipped Optical - OFDM (ACO-OFDM).

#### 3.2.2. Multiple Access

One of the core aspects of any positioning systems is the MT ability to distinguish reference measurements from the Anchors. As were illustrated in the taxonomy, there are several options to employ such as time division multiplexing, frequency division multiplexing, code division multiplexing, wavelength division multiplexing, and space division multiplexing. The benefits and drawbacks of the approaches are summarized in Table 2.

##### Time Division Multiplexing (TDM)

**Figure 5:**
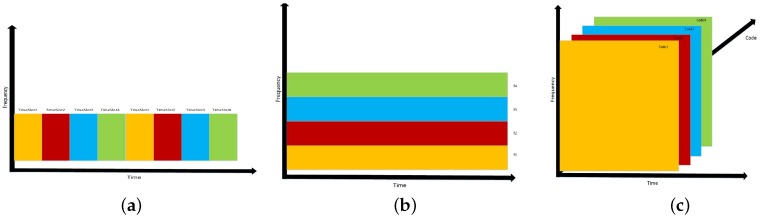
Model Description: (**a**) Time Division Multiplexing among 4 Timeslots; (**b**) Frequency Division Multiplexing among 4 Frequencies; (**c**) Code Division Multiplexing among 4 Spreading Codes.

Many systems and studies have employed a TDM scheme to segregate anchor signals. In such a scheme each anchor is allocated a time slot in which their transmission takes place. Figure 5a illustrates this concept using an example of 4 timeslots. Anchor 1 transmits in Timeslot 1 and does not transmit in Timeslots 2, 3, and, 4. This approach necessitates synchronization among all anchors such that the coordination can happen, or at a very minimum a collision avoidance scheme. Furthermore, one can observe that the downside to this is as the number of anchors grow, the latency in position updates will become large. An advantage, however, to TDM is the simplicity in the MT receiver design. Using TDM as a multiplexing scheme enables the use of TDoA, (ToF if the Anchors and MT are synchronized as well) RSS, and AoA measurements.

##### Frequency Division Multiplexing (FDM) & Wavelength Division Multiplexing (WDM)

FDM as opposed to TDM is an asynchronous multiplexing technique and does not require the anchors to coordinate their transmissions as each transmission is modulated onto a different frequency as illustrated in Figure 5b with four candidate transmission frequencies. The simplicity on the anchor side pushes the complexity to the MT ability to de-multiplex the signals from all anchors within its field of view. For the purpose of this article, OFDM is investigated as a form of FDM. One of the major downsides of OFDM is the effect of inter-symbol interference (ISI) due to transmission at high symbol rates, yet OFDM has an advantage that its use with an appropriate length cyclic prefix can enable high-rate communication over the visible light channel while providing asynchronous multiple access. FDM approaches enable scalability of the network as opposed to TDM approaches. Another perspective on FDM can be viewed as WDM, since frequency and wavelength are inversely proportional via the speed of light λ=c/f. In the optics field, color is conventionally specified in terms of wavelength λ. In terms of visible light positioning, anchor LEDs can be muti-chip e.g., Red Green Blue Yellow (RGBY), RGB, etc. These LEDs can be controlled to create different color renderings at the MT and the MT could be designed to have a receiver to de-multiplex the colors from each anchor; this is also an asynchronous multiplexing scheme. The aims of this article use blue phosphor LEDs as the Anchor nodes and as such WDM is not explored herein. For both FDM and WDM, without the requirement of synchronization being met, only RSS or AoA measurements may be obtained.

##### Code Division Multiplexing (CDM)

CDM is yet another asynchronous multiplexing approach which does not require major infrastructure investment in which unique codes are applied to each Anchor Luminaire. The MT must have knowledge of all the unique codes to uncover each code by transmitted by each Anchor. An illustration of the CDM is in Figure 5c. It should be noted that CDM has a rich library of codes with attractive auto correlation properties that have been explored in both RF communications (e.g., m-codes and Gold Sequences) and Unipolar Fiber Optic communications (e.g., Optical Orthogonal Codes and Prime Codes) RSS measurements can be calculated from the autocorrelation peak at the MT. AoA measurements can also be calculated using directional antenna arrays derived from RSS element measurements. An investigation of the effect on multiple access interference using such codes concluded that extended quadratic sequences, a type of prime code is best used for visible light positioning systems [40]. CDM is more robust to noise sources than either FDM or TDM, yet it suffers from multiple access interference (MAI). It has also been proposed that increasing the code length as well as the MT FoV can help to eliminate the MAI on positioning error [40]. CDM approaches as FDM enable a scalable network.

### 3.3. Fine Phase

The *fine* phase of the two-phase algorithm capitalizes on having multiple anchor luminaries in view to perform trilateration or triangulation at the MT. In this section different measurements are evaluated: AoA, ToF, and RSS. When AoA measurements are observed, a novel iterative triangulation algorithm is introduced based on azimuth and elevation angles constrained by the geometry of the MT-anchor geometry. In addition to this we expand the fine phase to include ToF and RSS measurements using least-squares triangulation. It is noted that other algorithms to perform triangulation or trilateration may be performed in the fine phase, but the key application is the framework that is introduced.

### 3.4. Fine Phase-Triangulation Based Positioning Using AoA

AoA is in general a direction finding technique, which focuses on the ability to measure angles between transmitters and receivers rather than distances. AoA finds application in scenarios where antennas are directional. If the *j*th receiver is equipped to measure AoA’s $\beta_{k}$ from up to *n* beacons. In general an over determined least squares solutions can be obtained.

(17) $$A=\left[\begin{array}{cc} -\sin\beta_{1} & \cos\beta_{1}\\ \vdots & \vdots \\ -\sin\beta_{n} & \cos\beta_{n} \end{array}\right]$$

(18) $$b=\left[\begin{array}{c} y_{1}\cos\beta_{1}-x_{1}\sin\beta_{1}\\ \vdots \\ y_{n}\cos\beta_{n}-x_{n}\sin\beta_{n} \end{array}\right]$$

(19) $$\hat{r_{j}^{mobile}}={\left(A^{T}A\right)}^{-1}A^{T}b$$

This work introduces a novel algorithm to use with AoA measurements. The orientation of the target is crucial and the determination of target location is geometry and search related given the information available. The *coarse* phase of the algorithm measured $P_{R_{k_{q},j}}\left(\phi_{k_{q},j},\theta_{k_{q},j}\right)$ and provided an initial guess of the range, ${∥{\hat{R}}_{k_{q},j}∥}_{2}=\left|\right|{\hat{r}}_{R_{j}}-r_{S_{k_{q,\mathrm{desired}}}}\left|\right|$ between the source Luminaire anchor and the MT. Therefore, in certain circumstances, we may assume that $\theta_{k_{q},j}=\phi_{k_{q},j},\forall k\in L,q\in Q,j\in M$. Under this assumption, we may compute an estimate for the angle of incidence, $\theta_{k,j}$.

(20) $$\theta_{k_{q},j}=\cos^{-1}\left[{\left(\frac{P_{R_{k_{q},j}}\left(\phi_{k_{q},j},\theta_{k_{q},j}\right)}{P_{R_{k_{q},j}}\left(0,0\right)}\right)}^{\frac{1}{m+M+\kappa }}\right]$$

**Figure 6:**
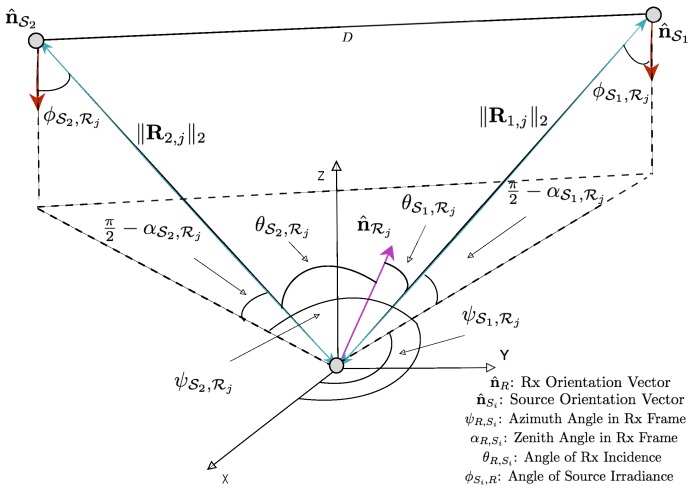
Two Source-Single Receiver Geometry Illustration for Azimuth and Elevation Angle of Arrival Measurement with Local Coordinate Frame.

The *fine* phase of the two-phase positioning algorithm served to improve the initial *coarse* estimate by measuring bearing and elevation angles observed from the landmark source Luminaries and triangulating its position. There are several approaches to triangulation in the literature such as iterative search, geometric circle intersection, geometric triangulation, and the Newton-Raphson method [41]. Firstly, to place the AoA analysis of bearing/azimuth and elevation discussion into context, give attention to the geometry outlined in Figure 6. To consider the geometry of the Luminaire anchor and MT pairs, ray optics may be applied to infer angular information. $LOS_{k_{q},j}$ is the line of sight vector of illumination from the *q*th LED within the *k*th Luminaire anchor to the *j*th MT; it has length ${∥R_{k_{q},j}∥}_{2}$ and orientation ${\hat{n}}_{S_{k_{q}}}$.

Typically three dimensional triangulation requires four reference anchors due to the fact that each Luminaire anchor provides one independent AoA measurement to the MT. However, if the MT is capable of measuring both Azimuth $\left({\hat{\psi }}_{k,j}\right)$ and Elevation $\left({\hat{\alpha }}_{k,j}\right)$ with respect to its orientation axis (e.g., the *z*-axis is ${\hat{n}}_{R_{j}}$), only two anchors are required for triangulation. Let $D_{x}$ and $D_{y}$ denote the inter-luminaire spacing in the *x* and *y* directions, mounted on the ceiling. Given these two distances any inter-anchor distance, *D*, can be computed when determining which anchors to use for triangulation. Furthermore, we may compute the ranges between the anchors k=1,2 and the *j*th receiver of interest as follows:

(21) $${∥{\hat{R}}_{1,j}∥}_{2}=\frac{1}{Q}\sum_{q=1}^{Q}\frac{D\sqrt{1+\tan^{2}{\hat{\psi }}_{1_{q},j}}}{\sin{\hat{\alpha }}_{1_{q},j}\left(\tan{\hat{\psi }}_{1_{q},j}\tan{\hat{\psi }}_{2_{q},j}\right)}$$

(22) $$D=\sqrt{D_{x}^{2}+D_{y}^{2}}$$

(23) $${∥{\hat{R}}_{2,j}∥}_{2}=\frac{1}{Q}\sum_{q=1}^{Q}\frac{D\sqrt{1+\tan^{2}{\hat{\psi }}_{2_{q},j}}}{\sin{\hat{\alpha }}_{2_{q},j}\left(\tan{\hat{\psi }}_{1_{q},j}\tan{\hat{\psi }}_{2_{q},j}\right)}$$

(24) $${\hat{r}}_{R_{j_{FINE}}}=\frac{1}{L}\sum_{k=1}^{L}\left(\left[\begin{array}{c} X_{k}\\ Y_{k}\\ Z_{k} \end{array}\right]+\left[\begin{array}{c} {∥{\hat{R}}_{k,j}∥}_{2}\sin{\hat{\alpha }}_{k,j}\cos{\hat{\psi }}_{k,j}\\ {∥{\hat{R}}_{k,j}∥}_{2}\sin{\hat{\alpha }}_{k,j}\sin{\hat{\psi }}_{k,j}\\ {∥{\hat{R}}_{k,j}∥}_{2}\cos{\hat{\alpha }}_{k,j} \end{array}\right]\right)$$

It can be seen in Equations (21), (23) and (24) that given perfect measurements of the Azimuth $\left({\hat{\psi }}_{k,j}=\psi_{k,j}\right)$ and Elevation $\left({\hat{\alpha }}_{k,j}=\alpha_{k,j}\right)$ between the MT and two anchor source Luminaries, the position of the MT can be analytically computed. Due to the non-linearity of the transformations, bias and precision errors in the angular measurements can drastically affect the estimate of MT position. There are measures to be taken to bound the effect instrumentation errors propagating. Given that lighting source Luminaries are typically installed in a fixed location within the indoor space, we may derive a geometric constraint [42] which aids in the removal of poor measurements. In this work, however, we assume that the light source Luminaries are on the ceiling as in Figure 1 and can derive the geometric constraint according to the illustration in Figure 6.

If one defines an error vector e as follows, for the azimuth and elevation errors for each anchor Luminaire observed by the MT over a measurement frame worth of time (e.g., 100 ms) samples.

(25) $$e={\left[e_{1},e_{2},e_{3},\ldots ,e_{M}\right]}^{T}$$

(26) $$e_{k}=\left[e_{\psi_{1,j}}\left[m\right],e_{\alpha_{1,j}}\left[m\right],e_{\psi_{2,j}}\left[m\right],e_{\alpha_{2,j}}\left[m\right]\right],m=1,2,\ldots ,M$$

(27) $$e_{\psi_{k,j}}\left[m\right]=\psi_{k,j}\left[m\right]-{\hat{\psi }}_{k,j}\left[m\right],m=1,2,\ldots ,M$$

(28) $$e_{\alpha_{k,j}}\left[m\right]=\alpha_{k,j}\left[m\right]-{\hat{\alpha }}_{k,j}\left[m\right],m=1,2,\ldots ,M$$

We propose to minimize the square error f(e) subject to the geometric constraint $c(e_{m})$ at each for each measurement during the measurement interval.

(29) $$\begin{array}{cccc} \underset{e}{\mathrm{argmin}} & f(e) & = & e^{T}e\\ \mathrm{subject}\mathrm{to} & c(e_{1}) & = & 0\\ & c(e_{2}) & = & 0\\ & c(e_{3}) & = & 0\\ & c(e_{4}) & = & 0\\ & & \vdots & \\ & c(e_{M}) & = & 0 \end{array}$$

We can observe that the constraint based on this configuration is given by:

(30) $$\begin{array}{cc} c(e_{m})= & \cot\left(\frac{\pi }{2}-{\hat{\alpha }}_{1,j}\left[m\right]-e_{\alpha_{1,j}}\left[m\right]\right)\sin\left({\hat{\psi }}_{1,j}\left[m\right]+e_{\psi_{1,j}}\left[m\right]-\frac{\pi }{2}\right)-\\ & \cot\left(\frac{\pi }{2}-{\hat{\alpha }}_{2,j}\left[m\right]-e_{\alpha_{2,j}}\left[m\right]\right)\sin\left(\frac{3\pi }{2}-{\hat{\psi }}_{2,j}\left[m\right]-e_{\alpha_{2,j}}\left[m\right]\right)=0 \end{array}$$

This constrained optimization problem may be solved with Lagrangian functions and may be transformed through linearly approximating the *M* constraint’s $c(e_{1})$, $c(e_{2})$, ..., $c(e_{M})$ into a more computationally effective Quadratic Programming (QP) problem [42].

(31) $$L=f\left(e\right)+\sum_{m=1}^{M}\lambda_{m}^{T}c\left(e_{m}\right)$$

Realizing this is in an iterative problem attempting to find to the optimal solution and Lagrange multipliers associated with the error vector $\left(e^{*},\lambda_{1}^{*},\lambda_{2}^{*},\lambda_{3}^{*},\ldots ,\lambda_{M}^{*}\right)$. Consider the *i*th iteration during the search for the optimal solution and let the current estimate of the Lagrange multipliers be $\left(e^{i},\lambda_{1}^{i},\lambda_{2}^{i},\lambda_{3}^{i},\ldots ,\lambda_{M}^{i}\right)$.

$$\begin{array}{cc} \underset{p}{\mathrm{argmin}} & \frac{1}{2}p^{i}H^{i}{\left(p^{i}\right)}^{T}+\nabla f\left(e^{i}\right){\left(p^{i}\right)}^{T}\\ \mathrm{subject}\;\mathrm{to} & \nabla c\left(e^{i}\right){\left(p^{i}\right)}^{T}+\sum_{m=1}^{M}c\left(e^{i}\right)=0 \end{array}$$

(32) $$e^{i}={\left[e_{1}^{i},e_{2}^{i},e_{3}^{i},\ldots e_{M}^{i}\right]}^{T}$$

(33) $$A=\left(\begin{array}{cccc} H^{i} & -\nabla c{\left(e_{1}^{i}\right)}^{T} & \cdots & -\nabla c{\left(e_{M}^{i}\right)}^{T}\\ \nabla c(e_{1}^{i}) & \vdots & \cdots & \vdots \\ \vdots & \vdots & \ddots & \vdots \\ \nabla c(e_{M}^{i}) & 0 & \cdots & 0 \end{array}\right)$$

(34) $$x=\left(\begin{array}{c} {\left(p^{i}\right)}^{T}\\ \mu_{1}^{i}\\ \vdots \\ \mu_{M}^{i} \end{array}\right)$$

(35) $$b=\left(\begin{array}{c} -\nabla L{\left(e^{i},\lambda_{1}^{i},\cdots ,\lambda_{M}^{i}\right)}^{T}\\ c(e_{1}^{i})\\ \vdots \\ c(e_{M}^{i}) \end{array}\right)$$

(36) Ax=b

The variables $p^{i}$ and $\mu_{1}^{i},\ldots ,\mu_{M}^{i}$ are the corrections applied to $e^{i}$ and $\lambda_{1}^{i},\ldots \lambda_{M}^{i}$ respectively if they satisfy the Wolfe Conditions in Equation (37).

(37) $$\left(\begin{array}{c} {\left(e^{i+1}\right)}^{T}\\ \lambda_{1}^{i+1}\\ \lambda_{2}^{i+1}\\ \lambda_{3}^{i+1}\\ \vdots \\ \lambda_{M}^{i+1} \end{array}\right)=\left(\begin{array}{c} {\left(e^{i}\right)}^{T}\\ \lambda_{1}^{i}\\ \lambda_{2}^{i}\\ \lambda_{3}^{i}\\ \vdots \\ \lambda_{M}^{i} \end{array}\right)+\alpha^{i}\left(\begin{array}{c} {\left(p^{i}\right)}^{T}\\ \mu_{1}^{i}\\ \mu_{2}^{i}\\ \mu_{3}^{i}\\ \vdots \\ \mu_{M}^{i} \end{array}\right)$$

The optimization is iterative and have pre-established performance criteria established such as an error tolerance (ζ≥0): $\left|\right|e_{i+1}-e_{i}\left|\right|\leq \zeta$ to prevent diminishing returns on computation or subject to a minimum of a maximum iteration constraint: $I_{max}$ or a computation time constraint $\tau_{max}$, to prevent excessive computation time. This personal work of the two-phase algorithm has been published [4].

(38) $$\tau =\min\left\{I_{max},\tau_{max}\right\}$$

### 3.5. Fine Phase-Trilateration Based Positioning Using ToF or RSS

In an attempt to provide a broader framework, other measurements are evaluated, yet in this work published trilateration algorithms are used as the positioning mechanism as opposed to the novel framework previously presented in the AoA case. Another means to obtain a distance measurement between anchor and mobile target is the through the ToF. The ToF of a transmitted signal is proportional to the distance traveled through the velocity of the signal (the speed of light, *c*). One of the main constraints is the coupling between clock synchronization and supporting such measurements [43].

It is when the synchronization of clocks occur that one may determine distance from target *j* to anchor *k*: $D(r_{j}^{mobile},r_{k})$, by the product of the propagation speed *c* and $ToF_{j,k}$. If we define the transmission time as $t_{k}$ and the received time $t_{j}$, then $ToF_{j,k}=\left(t_{j}-t_{k}\right)$

(39) $$D\left(r_{j}^{mobile},r_{k}\right)=c\cdot ToF_{j,k}$$

If the distance travelled is two-way (i.e. from the anchor to the target and back to the anchor), it is termed two-way ToF and is defined mathematically as:

(40) $$2D\left(r_{j}^{mobile},r_{k}\right)=c\cdot ToF_{j,k}$$

The RSS at the *j*th receiver, $P_{R,j}$, from the *k*th transmitter with transmit power $P_{T,k}$, is yet another observable that may be used to determine the location of a target. The basis of RSS is that the received signal power rolls off with the square of separation between the *k*th anchor and *j*th target, $D(r_{j}^{mobile},r_{k})$. In general, the squared distance is proportional to the ratio of transmitted power to received power via a proportionality factor χ.

(41) $$P_{R,j}=\frac{\chi P_{T,k}}{D{\left(r_{j}^{mobile},r_{k}\right)}^{2}}\Rightarrow D\left(r_{j}^{mobile},r_{k}\right)=\sqrt{\frac{\chi P_{T,k}}{P_{R,j}}}$$

The same general form applies for an RF channel and a VLC channel:

- RF Channel: $\chi =G_{T}G_{R}\frac{\lambda^{2}}{16\pi^{2}}$, where $G_{T}$ is the gain of the transmit antenna, $G_{R}$ is the gain of the receive antenna, λ is the wavelength of the RF signal.
- VLC Channel: $\chi =T\left(\phi_{k,j}\right)A_{R_{j}}g\left(\theta_{k,j}\right)$, where $T\left(\phi_{k,j}\right)$ angle of emission dependent transmit optics, $A_{R_{j}}$ is the effective area of the photodetector, $g\left(\theta_{k,j}\right)$ is the angle of incidence dependent concentration optics at the receiver.

(42) $$P_{R_{j}}=\frac{P_{T_{k}}T\left(\phi_{k,j}\right)A_{R_{j}}g\left(\theta_{k,j}\right)}{D{\left(r_{j}^{mobile},r_{k}\right)}^{2}}\Rightarrow D\left(r_{j}^{mobile},r_{k}\right)=\sqrt{\frac{P_{T_{k}}T\left(\phi_{k,j}\right)A_{R_{j}}g\left(\theta_{k,j}\right)}{P_{R_{j}}}}$$

(43) $$T\left(\phi_{k,j}\right)=\frac{m+1}{2\pi }\cos^{m}\left(\phi_{k,j}\right)$$

(44) $$g\left(\theta_{k,j}\right)=\left\{\begin{array}{cc} \frac{n^{2}}{\sin^{2}\left(\theta_{k,j}\right)}, & \mathrm{if}\;0\leq \theta_{k,j}\leq FOV\leq \frac{\pi }{2}\\ 0, & \mathrm{otherwise} \end{array}\right.$$

In particular, least squares triangulation based on overdetermined set of equations is used to map a sequence of ToF/Range or RSS/Range measurements into positional coordinates.

To arrive at a positional measurement from these ToF or RSS measurement derived distances, least squares trilateration based on overdetermined set of equations is employed (e.g., determining the intersection of spheres) [44,45] as an example positioning algorithm. Trilateration is a technique used to compute a position of an target *j*, given its distance measurements $D(r_{j}^{mobile},r_{0})$, $D(r_{j}^{mobile},r_{1})$, $D(r_{j}^{mobile},r_{2})$ in three dimensional Euclidean space $R^{3}$ from, at a minimum, three transmission sources. Trilateration gathers three (3) non-coplanar reference range measurements as follows:

(45) $$\begin{array}{c} {\left(x_{j}-x_{0}\right)}^{2}+{\left(y_{j}-y_{0}\right)}^{2}+{\left(z_{j}-z_{0}\right)}^{2}=D^{2}\left(r_{j}^{mobile},r_{0}\right)\\ {\left(x_{j}-x_{1}\right)}^{2}+{\left(y_{j}-y_{1}\right)}^{2}+{\left(z_{j}-z_{1}\right)}^{2}=D^{2}\left(r_{j}^{mobile},r_{1}\right)\\ {\left(x_{j}-x_{2}\right)}^{2}+{\left(y_{j}-y_{2}\right)}^{2}+{\left(z_{j}-z_{2}\right)}^{2}=D^{2}\left(r_{j}^{mobile},r_{2}\right) \end{array}$$

Typically this is solved via a least squares algorithm. The following matrices are defined such that $A{\hat{r}}_{j}^{m}=b$:

(46) $$A=2\left[\begin{array}{ccc} x_{n}-x_{1} & y_{n}-y_{1} & z_{n}-z_{1}\\ \vdots & \vdots & \vdots \\ x_{n}-x_{n-1} & y_{n}-y_{n-1} & z_{n}-z_{n-1} \end{array}\right]$$

(47) $$b=\left[\begin{array}{c} \left(r_{1}^{2}-r_{n}^{2}\right)-\left(x_{1}^{2}-x_{n}^{2}\right)-\left(y_{1}^{2}-y_{n}^{2}\right)-\left(z_{1}^{2}-z_{n}^{2}\right)\\ \vdots \\ \left(r_{n-1}^{2}-r_{n}^{2}\right)-\left(x_{n-1}^{2}-x_{n}^{2}\right)-\left(y_{n-1}^{2}-y_{n}^{2}\right)-\left(z_{n-1}^{2}-z_{n}^{2}\right) \end{array}\right]$$

(48) $${\hat{r}}_{R_{j_{FINE}}}={\hat{r}}_{j}^{m}={\left(A^{T}A\right)}^{-1}A^{T}b$$

## 4. Results

### 4.1. Coarse

**Figure 7:**
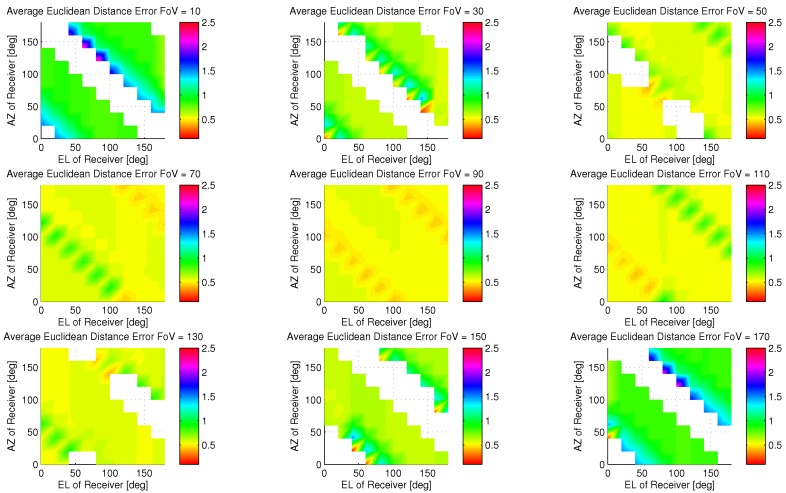
Coarse Performance for Varying Field of Views as a function of Receiver Orientation.

Firstly, the effect of orientation and field of view on the receiver design was studied to bound the design space; if the coarse performance is poor or numerically ill defined, the fine phase of the algorithm is of no use. Figure 7 illustrates the mean Euclidean distance error as an intensity plot for fixed field of views as a function of receiver orientation (Azimuth and Elevation in its local coordinate frame).

It is observed that for FoV∉[70,110], there are potential orientation configurations in which a coarse estimate cannot be made (as seen as white patches in Figure 7). The 90 degree FoV provides the best mean Euclidean distance accuracy performance over all receiver orientation ranging from 42 cm to 55 cm when not being directly beneath luminaries. Taking this result into account, the spatial error distribution is investigated for the coarse phase of the algorithm. The intensity map of accuracy error is shown in Figure 7. The largest spatial contributors of error are the poorly illuminated areas; however occupants are generally not isolated to these corner areas. We can observe that the coarse phase effectively groups the receiver to the closest luminaries or combination thereof, therefore the performance of this scheme is best when the receiver is in the closest proximity to luminaries.

#### 4.1.1. Coarse Phase-Proximity-Based Positioning with Time Division Multiplexing

As discussed in Section 3.2.1, the modulations that are being employed are OOK as a reference, 2-PPM as a less bandwidth efficient modulation relative to OOK, and 4-PAM as a less power efficient modulation relative to OOK using IM-DD over the visible light channel. By using TDM as the multiplexing scheme among each of the 12 anchor luminaries as depicted in Figure 1, the coarse phase of the algorithm is employed at each of the 169 discrete locations. The positioning performance is supplied over Monte Carlo simulation for 1200 trials for each modulation. Table 3 illustrates the choice of modulation doesn’t affect the performance of the coarse phase positioning when using TDM as the multiplexing scheme at the anchor luminaries.

**Figure 8:**
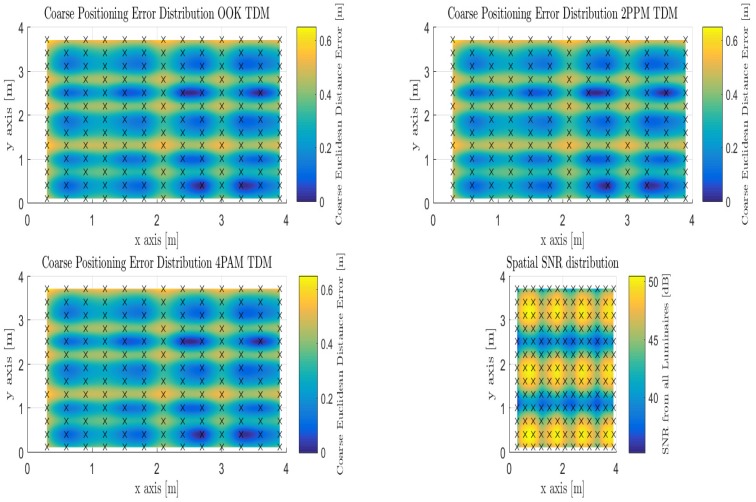
Illustration of Coarse Proximity-based Positioning with Time Division Multiplexing for OOK, 2-PPM, and 4-PAM pulsed modulations under the Spatial SNR Room Distribution.

It was decided to present the mean, median, mode, and standard deviation of the positioning error due to the spatial discrepancies across the candidate indoor environment. It can be observed that the mode positioning error over the entire indoor space is sub-centimeter (e.g., 0.65 cm). The majority of the room is fully illuminated with sufficient optical power to produce adequate positioning, however, in looking at the mean value of the positioning error we observe that it increases to 31.5 cm and a median of 31.49 cm. Both of which can account for the less illuminated areas throughout the room. Figure 8 shows the spatial distribution of SNR overlaid throughout the room at the 2.2 m reference height. Each of the error distribution maps depict the correlation with optical power strength with the positioning performance as described in Equation (13).

#### 4.1.2. Coarse Phase-Proximity-Based Positioning with Frequency Division Multiplexing

The modulations that are used to enable frequency division multiplexing were ACO-OFDM and DCO-OFDM. There is a trade between ACO-OFDM and DCO-OFDM in terms of its effect on BER. ACO-OFDM is more power-efficient than DCO-OFDM due to not having a DC-bias and bandwidth inefficient compared to DCO-OFDM due to selective use of subcarriers on which to transmit. The fact that illumination levels within the indoor space produce high signal to noise ratios spatially throughout the indoor space, the differences between ACO-OFDM and DCO-OFDM are not observed in the coarse phase of the algorithm. The positioning performance is supplied over Monte Carlo simulation for 1200 trials for each of the modulations.

**Figure 9:**
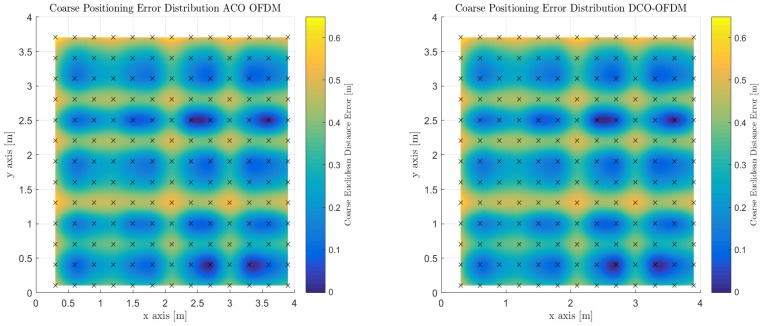
Illustration of Coarse Proximity-based Positioning with Frequency Division Multiplexing for DCO-OFDM and ACO-OFDM modulations under the Spatial SNR Room Distribution.

Figure 9 depicts the performance spatially of the OFDM schemes over the entire indoor space and they perform the same, which is expected at high signal to noise ratios. Table 4 again illustrates the mean, median, mode and standard deviations of the using OFDM as a multiplexing scheme during the coarse phase of positioning. It does perform slightly better on all accounts than synchronized TDM. Median positioning error is 30.29 cm.

#### 4.1.3. Coarse Phase-Proximity-Based Positioning with Code Division Multiplexing

Akin to the TDM evaluation, the modulations being used are also pulsed. OOK is used as a reference, 2-PPM as a less bandwidth efficient modulation relative to OOK, and 4-PAM as a less power efficient modulation relative to OOK using IM-DD over the visible light channel. By using CDM as the multiplexing scheme among each of the 12 anchor luminaries as depicted in Figure 1, the coarse phase of the algorithm is employed at each of the 169 discrete locations, but the principle difference compared to TDM is the presence of multiple access interference (MAI). It has been shown that the use of extend quadratic sequences, a type of prime code, is best used for indoor positioning using visible light compared to other families of codes used in RF and Fiber communications. The positioning performance is supplied over Monte Carlo simulation for 1200 trials for each modulation. Table 5 illustrates the choice of modulation affects the performance of the coarse phase positioning when using CDM as the multiplexing scheme at the anchor luminaries.

**Figure 10:**
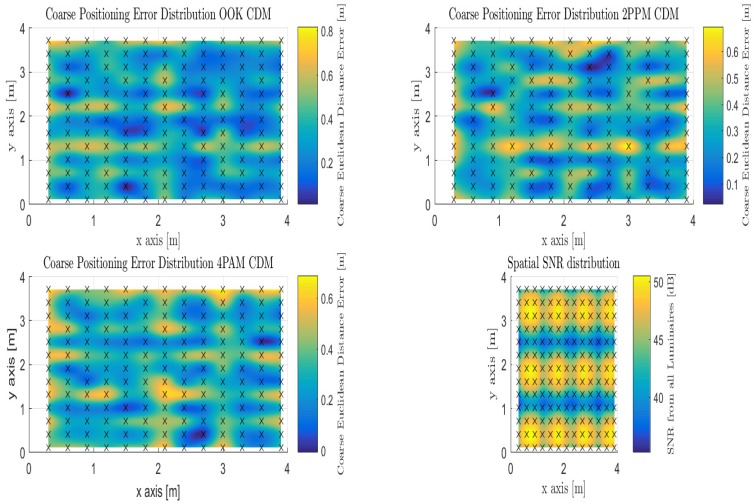
Illustration of Coarse Proximity-based Positioning with Code Division Multiplexing for OOK, 2-PPM, and 4-PAM pulsed modulations under the Spatial SNR Room Distribution.

It can be observed that the mode positioning error over the entire indoor space varies per pulsed modulation. The majority of the room is fully illuminated with sufficient optical power to produce adequate positioning, however, in looking at the mean value of the positioning error we observe that it increases again varying with modulation from (OOK: 34.06 cm, 2-PPM: 34.04 cm, and 4-PAM: 33.21 cm) and a median of (OOK: 32.58 cm, 2-PPM: 34.78 cm, and 4-PAM: 32.05 cm). All of which can account for the less illuminated areas throughout the room impacting the position estimate accuracy throughout the indoor environment. Each of the error distribution maps in Figure 10 depict the correlation with optical power strength with the positioning performance as described in Equation (13).

One can observe that there is still a concentrated clustering around each of the Luminaries on the outer-edges of the candidate environment, while in between Luminaries the coarse estimate is weighted in between those which are within the field of view while using CDM as occurred with OFDM and TDM. The difference is that the MAI add more distortion to the positional estimate for each of the evaluated modulations.

**Figure 11:**
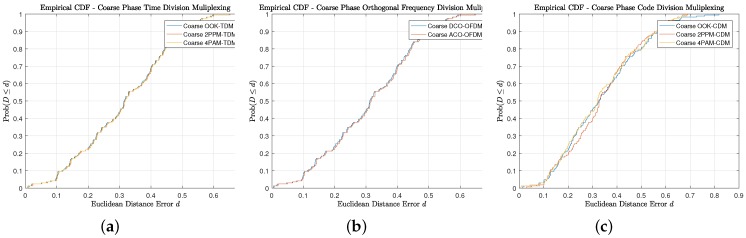
Model Description: (**a**) Empirical CDF of Euclidean Distance Error for Time Division Multiplexing using Coarse Phase Proximity Positioning; (**b**) Empirical CDF of Euclidean Distance Error for Orthogonal Frequency Division Multiplexing using Coarse Phase Proximity Positioning; (**c**) Empirical CDF of Euclidean Distance Error for Code Division Multiplexing using Coarse Phase Proximity Positioning.

The precision of the approach is inspected, Figure 11 illustrates the cumulative distribution function (CDF) and it is directly apparent that the reported accuracy numbers are at the fiftieth percentile, while the ninetieth percentile are again modulation dependent. (OOK: 57.5 cm, 2-PPM: 56.5 cm, and 4-PAM: 56.3 cm). The modulation dependency, qualitatively, is not a substantial factor in the coarse error distribution throughout the candidate indoor environment as the multiple access interference dominates and provides degradation relative to the synchronous TDM multiplexing approach or the asynchronous OFDM multiplexing approach.

In comparing the three multiplexing scheme’s performance during the Coarse Phase in Table 6, it is noted that OFDM performs the best of all three schemes yet it does require more sophisticated modulation. In terms of pulsed modulation scheme’s TDM outperforms CDM.

### 4.2. Fine

The fine phase of the algorithm serves to improve the initial coarse estimate. The Euclidean distance error is improved over the interior of the room when compared to the coarse phase performance as the receiver is able to arrive at useful independent measurements and can refine the initial coarse estimate, if sufficient anchor luminaries are in view. It is noticed, however, that the fine phase processing has little impact on the poorly illuminated sections of the room. The results reported offer significant improvements over state of the art piggyback approaches due to both the ubiquity and distribution of anchor sources provided throughout the indoor environment as well as the directionality of the medium over which the positioning is performed. Moreover the computational complexity is low as a coarse estimate is found within one multiple access cycle, whereas the fine estimate arrives at a solution within four iterations on average. This algorithm is performed in the mobile device and can be thought of as distributed; however as we have shown herein the device may report its estimates of its location to the infrastructure to allow the infrastructure to provide services or resources to the mobile device.

Table 7 illustrates the average, median, and standard deviation of the fine phase performance for the six (6) evaluated transmission multiplexing and measurement/positioning schemes. It must be noted, however, that the only valid areas within the indoor environment contribute to the statistics reported. OFDM using RSS performs the best with TDM using ToF performing slightly worse, but at the expense of synchronization for TDM ToF, OFDM RSS appears much more attractive.

**Figure 12:**
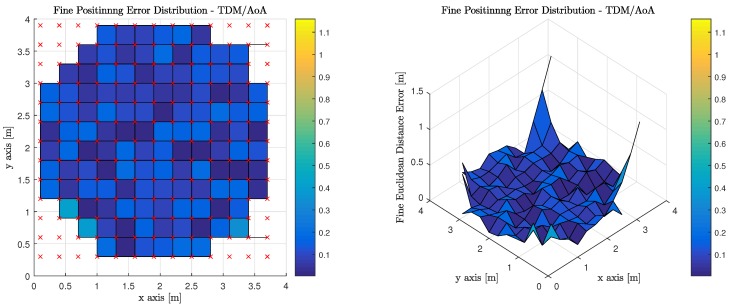
Fine Phase Positioning Performance in Candidate Room using Angle of Arrival Measurement, Geometric Constrained Optimized Triangulation Positioning Algorithm and Time Division Multiplexing.

Figure 12 illustrates the performance of using AoA measurements to perform geometric constrained triangulation when the observations from the MT are made on anchor Luminaries, which have access to the visible light channel via TDM. It is worth noting, that red ‘x’s denote the true position of the MT at each of the discrete location’s the computations are performed. The corners of the room cannot return a valid positional estimate because an insufficient number of anchor luminaries are in the MT’s FoV.

**Figure 13:**
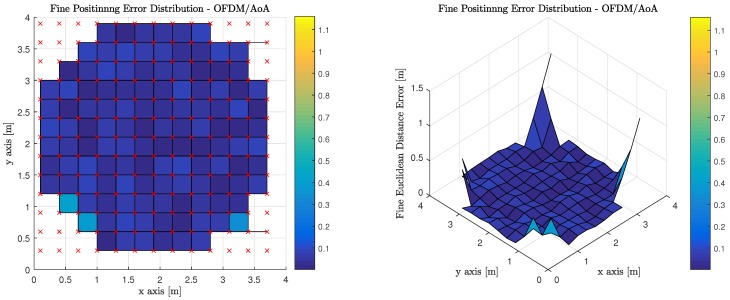
Fine Phase Positioning Performance in Candidate Room using Angle of Arrival Measurement, Geometric Constrained Optimized Triangulation Positioning Algorithm and Orthogonal Frequency Division Multiplexing.

Figure 13 illustrates the performance of using AoA measurements to perform geometric constrained triangulation when the observations from the MT are made on anchor Luminaries, which have access to the visible light channel via OFDM. It is worth noting, that red ‘x’s denote the true position of the MT at each of the discrete location’s the computations are performed. The corners of the room cannot return a valid positional estimate because an insufficient number of anchor luminaries are in the MT’s FoV.

**Figure 14:**
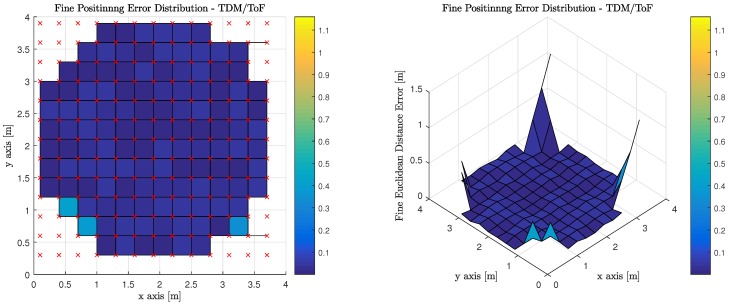
Fine Phase Positioning Performance in Candidate Room using Time of Flight Measurement, Geometric Constrained Optimized Triangulation Positioning Algorithm and Time Division Multiplexing.

Figure 14 illustrates the performance of using ToF measurements to perform least squares trilateration when the observations from the MT are made on anchor Luminaries, which have access to the visible light channel via TDM. It is worth noting, that red ‘x’s denote the true position of the MT at each of the discrete location’s the computations are performed. The corners of the room cannot return a valid positional estimate because an insufficient number of anchor luminaries are in the MT’s FoV.

On top of the ToF measurement, the RSS measurement is evaluated. Again, least squares trilateration based on overdetermined set of equations is used to map a sequence of RSS/Range measurements into positional coordinates as with ToF measurements defined above.

**Figure 15:**
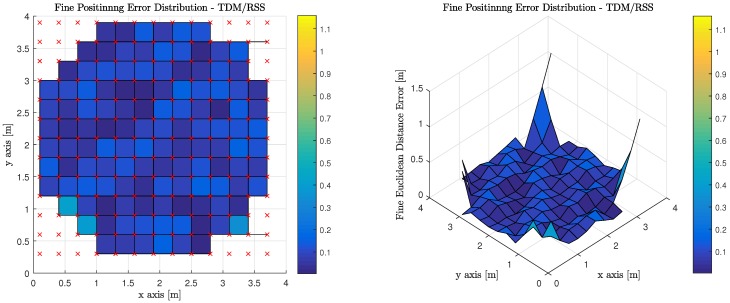
Fine Phase Positioning Performance in Candidate Room using Received Signal Strength Measurement, Least Squares Trilateration Positioning Algorithm and Time Division Multiplexing.

Figure 15 illustrates the performance of using RSS measurements to perform least squares trilateration when the observations from the MT are made on anchor Luminaries, which have access to the visible light channel via TDM. It is worth noting, that red ‘x’s denote the true position of the MT at each of the discrete location’s the computations are performed. The corners of the room cannot return a valid positional estimate because an insufficient number of anchor luminaries are in the MT’s FoV.

**Figure 16:**
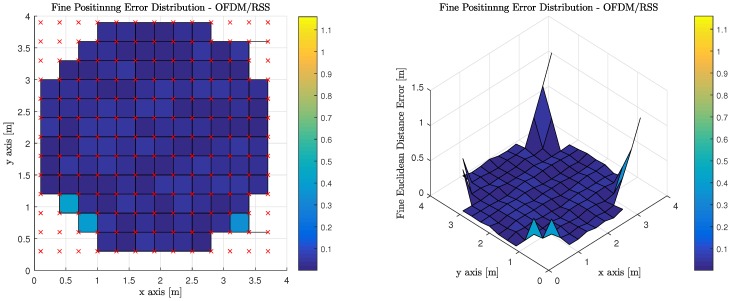
Fine Phase Positioning Performance in Candidate Room using Received Signal Strength Measurement, Least Squares Trilateration Positioning Algorithm and Orthogonal Frequency Division Multiplexing.

Figure 16 illustrates the performance of using RSS measurements to perform least squares trilateration when the observations from the MT are made on anchor Luminaries, which have access to the visible light channel via OFDM. It is worth noting, that red ‘x’s denote the true position of the MT at each of the discrete location’s the computations are performed. The corners of the room cannot return a valid positional estimate because an insufficient number of anchor luminaries are in the MT’s FoV.

**Figure 17:**
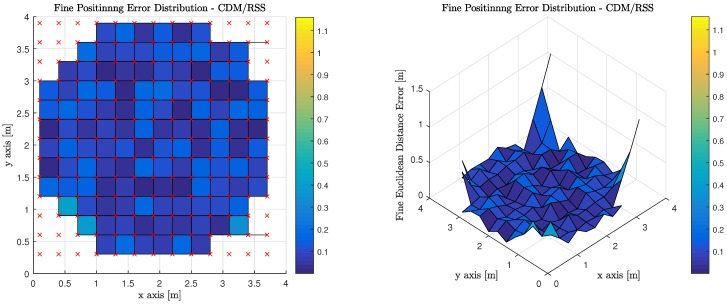
Fine Phase Positioning Performance in Candidate Room using Received Signal Strength Measurement, Least Squares Triangulation Positioning Algorithm and Code Division Multiplexing.

Figure 17 illustrates the performance of using RSS measurements to perform geometric constrained triangulation when the observations from the MT are made on anchor Luminaries, which have access to the visible light channel via CDM. It is worth noting, that red ‘x’s denote the true position of the MT at each of the discrete location’s the computations are performed. The corners of the room cannot return a valid positional estimate because an insufficient number of anchor luminaries are in the MT’s FoV.

### 4.3. Two-Phase

In general a Coarse phase positional estimate is always computed and stored away in memory so long as at least a single anchor Luminaire is within the MT’s FoV. As we have illustrated the coarse phase amounts to a weighted proximity estimate based on received optical power and anchor luminaire location and orientation. The Fine phase is also computed and stored away in memory if there are at least *N* anchor luminaries within the MT’s FoV. The problem with the fine phase by itself is that a positional estimate is not always produced and when the anchor requirement is not met, nothing is returned. This distorts the concept of statistical performance slightly insofar as the statistics discount the areas within the room that do not return an estimate (e.g., ‘NaN’).

The combination of Modulation, Coarse multiplexing, and fine channel measurement/fine positioning algorithms evaluated using the two-phase framework are illustrated in Table 8. The Two Phase approach, like the coarse phase, reports results over the entirety of the indoor space, contrary to the fine phase; this is the advantage to the scheme. The disadvantage, at first glance at the results, could be interpreted that the positioning performance has degraded.

**Figure 18:**
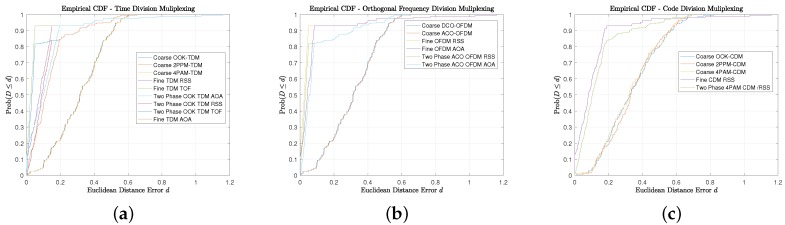
Model Description: (**a**) Two-Phase Precision with TDM Multiplexing; (**b**) Two-Phase Precision with OFDM Multiplexing; (**c**) Two-Phase Precision with CDM Multiplexing.

This is misleading, as the expanded footprint of the room coverage provides valid proximity position estimates, which affect average and median values. This can be observed in all the empirical CDFs in Figure 18 in which the Coarse phase, Fine Phase, and Combined Two-Phase precisions are presented. The general trend with all of the scenarios is that the coarse phase has the least sharp monotonic response as the positioning errors can be bounded by the largest footprint. Furthermore, it converges to probability 1 at the smallest distance. The phase has the sharpest monotonic behavior, which is sensible as a majority of the indoor environment has sufficient anchors in the MTs FoV, but the downside is that it coverages to probability 1 at the largest distance. The two-phase approach yields balance between the benefits of the coarse and the fine.

## 5. Conclusions

The complexity of the two-phase algorithm is balanced when compared to conventional approaches to the indoor positioning problem; its complexity is largely a function of the choices the designer makes for its application. ToF-based trilateration is typically complex in that it requires clock synchronization between anchors and MTs. AoA is typically complex in that it requires antenna arrays or an imaging receiver. RSS is typically less complex compared to the other two measurement types. Overlaid on this is the multiplexing and modulation schemes, which all have their own respective attractive and less desirable qualities. The advantage of the two-phase algorithm is that the complexity can be as low as one desires it to be (e.g., OOK/CDM/RSS/Trialteration) or as high as one desires (e.g., OFDM/AoA/Geometric Constrained Triangulation). In line with this IPS evaluation criteria, the complexity of this algorithm can be Low, Medium, or High. As was presented in the performance of the fine phase of the two-phase algorithm, when sufficient anchors aren’t within the FoV a positional estimate is not returned. The fine-phase is typically the only phase that occurs in indoor positioning systems published in the state of the art. The advantage of the coarse phase being prepended to the fine phase is that as long as a single anchor is in view, a positional estimate is returned. Furthermore, it is bounded by the illumination radius of the anchor within the MT’s FoV. Outlier suppression and blockage condition handling add to the robustness of the two phase framework. In line with this IPS evaluation criteria, the robustness of this algorithm can be High. This framework utilizes an infrastructure exploiting approach (IEA), which implies piggybacking upon the lighting and communication infrastructure. The modification to expand the footprint over which the positioning can apply changes when the luminaire anchor backbone network is updated. This is not necessarily non-zero work to expand the footprint, but in theory the majority of the effort would be to expand the lighting and communication services with positioning as a less of an impact overlay. In line with this IPS evaluation criteria, the scalability of this algorithm can be Medium and the expense of this algorithm can be Low since the major expense is the primary function of illumination not the overlaid services.

## Figures and Tables

**Table 1:** Two Phase Algorithm Parameters.

Parameter	Value	Units
Optical Tx Power $\left(P_{T}\right)$	1.924	W
Lambertian Mode (m)	1	-
Effective Area of Rx $\left(A_{R}\right)$	0.81	${\mathrm{cm}}^{2}$
Electron Charge (q)	$1.6\times 10^{-19}$	C
Background Light Current $\left(I_{BG}\right)$	$5100\times 10^{-6}$	A
Speed of Light (c)	$3.0\times 10^{8}$	m/s
Noise PSD $\left(N_{0}\right)$	$1.632\times 10^{-21}$	W/Hz
Optical / Electrical Efficiency $\left(R_{OE}\right)$	0.53	-
Source Orientation Vector ()	${\left[0,0,-1\right]}^{T}$	-
Rx Azimuth Angle ()	0−π	rad
Rx Elevation Angle ()	0−π	rad
Rx Orientation Vector ()	${\left[0,0,1\right]}^{T}$	
Vertical Range $\left(z_{k}-z_{j}\right)$	2.2	m
Symbol Rate $\left(R_{S}\right)$	20	symbols/s
Source Bandwidth (B)	20	MHz
Field of View (FoV)	0−π	rad
Wall Reflectivity $\left(\eta_{R}\right)$	0.6	-

**Table 2:** Benefits and Drawbacks of Multiplexing Approaches during Coarse Phase Positioning.

Coarse Phase Multiplexing Scheme	Benefits	Drawbacks
OFDM	Better suited for high data rate communication, Asynchronous	Complex Modulator and Demodulator
TDM	Simplistic	Requires Network Infrastructure, Highly Accurate Clocks, Synchronous
CDM	Asynchronous	Multiple Access Interference, Codes are not fully orthogonal

**Table 3:** Coarse Phase Positioning Performance for Pulsed Modulations using Time Division Multiplexing.

Pulsed Modulation Using TDM	Mean Positioning Error [m]	Median Positioning Error [m]	Mode Positioning Error [m]	Standard Deviation Positioning Error [m]
OOK	0.315	0.3149	0.0065	0.1437
2PPM	0.315	0.3149	0.0065	0.1437
4PAM	0.315	0.3149	0.0065	0.1437

**Table 4:** Coarse Phase Positioning Performance for Multicarrier Modulations using Orthogonal Frequency Division Multiplexing.

Multicarrier Modulation Using OFDM	Mean Positioning Error [m]	Median Positioning Error [m]	Mode Positioning Error [m]	Standard Deviation Positioning Error [m]
ACO-OFDM	0.305	0.3029	0.0056	0.1232
DCO-OFDM	0.305	0.3029	0.0056	0.1232

**Table 5:** Coarse Phase Positioning Performance for Pulsed Modulations using Code Division Multiplexing.

Pulsed Modulation Using CDM	Mean Positioning Error [m]	Median Positioning Error [m]	Mode Positioning Error [m]	Standard Deviation Positioning Error [m]
OOK	0.3406	0.3258	0.0121	0.1643
2PPM	0.3404	0.3278	0.0285	0.1515
4PAM	0.3321	0.3205	0.0095	0.1578

**Table 6:** Comparison among TDM, CDM, and OFDM in Coarse Phase.

Coarse Phase Multiplexing Scheme	Mean Pos. Error [m]	Median Pos. Error [m]	Mode Pos. Error [m]	Std Dev. Pos. Error [m]	90 Percent Precision
OFDM-ALL	0.305	0.3029	0.0056	0.1232	0.498
TDM-ALL	0.315	0.3149	0.0065	0.1437	0.5085
CDM-4PAM	0.3321	0.3205	0.0095	0.1578	0.563
CDM-2PPM	0.3404	0.3278	0.0285	0.1515	0.565
CDM-OOK	0.3406	0.3258	0.0121	0.1643	0.575

**Table 7:** Fine Phase Positioning: Average, Median, and Standard Deviation of Positioning Error.

Performance Metric	Average Positioning Error [m]	Median Positioning Error [m]	Std Dev Positioning Error [m]
**CDM with RSS**	0.1142	0.0931	0.1050
**OFDM with AOA**	0.0778	0.0422	0.1191
**OFDM with RSS**	0.0608	0.0260	0.1252
**TDM with AOA**	0.1213	0.0985	0.1014
**TDM with RSS**	0.1059	0.0803	0.1067
**TDM with TOF**	0.0616	0.0276	0.1249

**Table 8:** Two Phase: Coarse/Fine Positioning Average, Median, and Standard Deviation of Positioning Error.

Performance Metric	Average Positioning Error [m]	Median Positioning Error [m]	Std Dev Positioning Error [m]
**CDM with RSS**	0.1413	0.0997	0.0704
**OFDM with AOA**	0.0988	0.0452	0.0790
**OFDM with RSS**	0.0826	0.0328	0.0880
**TDM with AOA**	0.1403	0.1207	0.0539
**TDM with RSS**	0.1254	0.0975	0.0627
**TDM with TOF**	0.0833	0.0309	0.0877
